# Author Correction: Liquid flow reversibly creates a macroscopic surface charge gradient

**DOI:** 10.1038/s41467-021-27209-4

**Published:** 2021-11-24

**Authors:** Patrick Ober, Willem Q. Boon, Marjolein Dijkstra, Ellen H. G. Backus, René van Roij, Mischa Bonn

**Affiliations:** 1 Department of Molecular Spectroscopy, Max Planck Institute for Polymer Research, Mainz, Germany; 2 Institute for Theoretical Physics, Utrecht University, Utrecht, Netherlands; 3 Soft Condensed Matter, Debye Institute for Nanomaterials Science, Utrecht University, Utrecht, Netherlands; 4 Department of Physical Chemistry, University of Vienna, Vienna, Austria

**Keywords:** Geochemistry, Optical spectroscopy, Surface spectroscopy, Fluid dynamics

Correction to: *Nature Communications* 10.1038/s41467-021-24270-x, published online 2 July 2021.

The original version of this Article contained an error in Equation 8.

The second line of this equation omitted the multiplication factor 4 in the second term, and incorrectly read:

$$\frac{{\bar{\rho }}_{{{{{{\rm{F}}}}}}}(x)}{{\rho }_{{{{{{\rm{F}}}}}},{{{{{\rm{b}}}}}}}}=\left\{\begin{array}{ll}1+\varDelta {\rho }_{{{{{{\rm{F}}}}}}}\,(1-\frac{{x}^{2}}{{L}^{2}}) & {{{\rm{if}}}}\,{{{\rm{Pe}}}}\ll 1;\\ 1+\frac{\Delta {\rho }_{{{{{{\rm{F}}}}}}}}{{{{{{\rm{Pe}}}}}}}(1+\frac{x}{L}) & {{{\rm{if}}}}\,{{{\rm{Pe}}}}\gg 1\end{array}\right..(8)$$

The correct form of Equation 8 is:

8 $$\frac{{\bar{\rho }}_{{{{{{\rm{F}}}}}}}(x)}{{\rho }_{{{{{{\rm{F}}}}}},{{{{{\rm{b}}}}}}}}=\left\{\begin{array}{cc}1+\varDelta {\rho }_{{{{{{\rm{F}}}}}}}\,(1-\frac{{x}^{2}}{{L}^{2}}) & {{{\rm{if}}}}\,{{{\rm{Pe}}}}\ll 1;\\ 1+4\frac{\Delta {\rho }_{{{{{{\rm{F}}}}}}}}{{{{{{\rm{Pe}}}}}}}(1+\frac{x}{L}) & {{{\rm{if}}}}\,{{{\rm{Pe}}}}\gg 1\end{array}\right..$$

This has been corrected in the PDF and HTML versions of the Article.

The original version of the Supplementary Information associated with this Article contained an error in Supplementary Equation 17.

The equation omitted a factor 1/2 in front of the Peclet number Pe, and incorrectly read:

$$\frac{\rho (x)}{{\rho }_{{{{{{\rm{b}}}}}}}}=1+\frac{\Delta {\rho }_{{{{{{\rm{max }}}}}}}}{{{{{{\rm{Pe}}}}}}}\,\left(\frac{2x}{L}+\big(1+{e}^{2{{{{{\rm{Pe}}}}}}}-2{e}^{{{{{{\rm{Pe}}}}}}(1+\frac{x}{L})}\big)({{{{\mathrm{Coth}}}}}({{{{{\rm{Pe}}}}}})-1)\right)$$

The correct form of Supplementary Equation 17 is:

$$\frac{\rho (x)}{{\rho }_{{{{{{\rm{b}}}}}}}}=1+2\frac{\Delta {\rho }_{{{{{{\rm{max }}}}}}}}{{{{{{\rm{Pe}}}}}}}\,\left(\frac{2x}{L}+(1+{e}^{{{{{{\rm{Pe}}}}}}}-2{e}^{\frac{{{{{{\rm{Pe}}}}}}}{2}(1+\frac{x}{L})})({{{{\mathrm{Coth}}}}}\left(\frac{{{{{{\rm{Pe}}}}}}}{2}\right)-1)\right)$$

The original version of the Supplementary Information associated with this Article also contained an error in the 14^th^ sentence of the 6^th^ paragraph of Supplementary Discussion 2, where the Peclet number was incorrectly defined as ${{{{{\rm{Pe}}}}}}=2uL/D$. The correct version states ${{{{{\rm{Pe}}}}}}=2\bar{u}L/D$ in place of ${{{{{\rm{Pe}}}}}}=2uL/D$.

The HTML has been updated to include a corrected version of the Supplementary Information.

## Supplementary information

Updated Supplementary Information

