# Supplementary material for: Liquid flow reversibly creates a macroscopic surface charge gradient
Source: Nat Commun. 2021 Jul 2;12:4102. doi: 10.1038/s41467-021-24270-x (PMC8253779; doi:10.1038/s41467-021-24270-x)
Supplement: Supplementary file 1 — Supplementary Information [file 41467_2021_24270_MOESM1_ESM.pdf]

*Supplementary Information on*

# Liquid Flow Reversibly Creates a Macroscopic Surface Charge Gradient

Patrick Ober[1], Willem Q. Boon[2], Marjolein Dijkstra[3], Ellen H. G. Backus[1,4], René van Roij[2*] & Mischa Bonn[1*]

[1]Department of Molecular Spectroscopy, Max Planck Institute for Polymer Research, Ackermannweg 10, 55128 Mainz, Germany. [2]Institute for Theoretical Physics, Utrecht University, Princetonplein 5, 3584 CC Utrecht, Netherlands. [3]Soft Condensed Matter, Debye Institute for Nanomaterials Science, Utrecht University, Princetonplein 5, 3584 CC Utrecht, Netherlands. [4]Department of Physical Chemistry, University of Vienna, Waehringer Strasse 42, 1090 Vienna, Austria.

These authors contributed equally: Patrick Ober, Willem Q. Boon

*email: r.vanroij@uu.nl, bonn@mpip-mainz.mpg.de

## Supplementary Note 1.

## Details on v-SFG and how it reports on the surface charge

Within the dipole approximation, incident light induces a dipole moment, which on a macroscopic scale (averaging over many molecules), is the polarization. For weak incident fields, the induced dipole moment ($\mu$) or polarization ($P$) scales linearly with the electric field. However, for laser pulses also higher-order terms need to be taken into account. For the second- and third-order terms, the proportionality constants between the applied field and the induced dipole are the first- and second-order hyperpolarizability $\beta$ and $\gamma$, respectively. On the macroscopic, polarization scale, this is the second and third-order nonlinear susceptibility $\chi^{(2)}$ and $\chi^{(3)}$[1]

$$\begin{gathered}\mu = \mu_0 + \alpha E + \beta E^2 + \gamma E^3 + \cdots \\ P = P^{(1)} + P^{(2)} + P^{(3)} + \cdots = \epsilon_0\left(\chi^{(1)}E + \chi^{(2)}E^2 + \chi^{(3)}E^3 + \cdots\right).\end{gathered} \tag{S1}$$

The total electric field is the sum of the electric fields of the two incident laser pulses

$$E = E_{\mathrm{vis}}\cos(\omega_{\mathrm{vis}} \cdot t) + E_{\mathrm{IR}}\cos(\omega_{\mathrm{IR}} \cdot t), \tag{S2}$$

Where the amplitudes are given by $E_{vis}$ and $E_{IR}$ and the frequencies by $\omega_{vis}$ and $\omega_{IR}$. From combining equations (S2) and (S3), a second-order term with the sum of the incident frequencies arises[1]

$$P_{\mathrm{SFG}}^{(2)} = \epsilon_0\chi^{(2)}E_{\mathrm{vis}}E_{\mathrm{IR}}\cos\left((\omega_{\mathrm{vis}} + \omega_{\mathrm{IR}}) \cdot t\right). \tag{S3}$$

Considering that the intensity of the sum-frequency light scales with the square of the polarization, we can derive the relation[1-3]

$$I_{\mathrm{SFG}} \sim E_{\mathrm{vis}}^2 E_{\mathrm{IR}}^2 \left|\chi^{(2)}\right|^2, \quad \text{(S4)}$$

where $\chi^{(2)}$ is the macroscopic average of the first-order hyperpolarizability. In the case of a centrosymmetric system, $\chi^{(2)}$ becomes zero since the individual molecular hyperpolarizabilities cancel each other out, explaining the interface-specificity of v-SFG. At the interface, it is also crucial that a net-orientation of the molecules is present. In the case of random orientation, there is again canceling of the individual contributions. For the mineral-water interface, a net-orientation of interfacial water molecules is achieved by the presence of a surface charge.[1,2,4,5]

The presence of surface charge means that there is also an additional electric field present, which polarizes into the bulk and thereby breaks the symmetry requiring the addition of a $\chi^{(3)}$ contribution. The static, *DC* electric field determines the magnitude of the $\chi^{(3)}$ contribution[5-8]

$$I_{\mathrm{SFG}} \sim E_{\mathrm{vis}}^2 E_{\mathrm{IR}}^2 \left|\chi^{(2)} + \chi^{(3)} \int_0^\infty E_{\mathrm{DC}}(z)\,\mathrm{d}z\right|^2. \quad \text{(S5)}$$

We note that $\chi^{(2)}$ in Equation (S5) is effectively integrated over the interfacial layer where $\chi^{(2)}$ is non-zero. Within the Gouy-Chapman model, the electric field decays into the bulk solution as described by the Debye length. The Debye length is shorter with increasing salt concentration.[5-7]

Our measurements are performed in total internal reflection geometry, which is known to enhance the detected signal by orders of magnitude.[9] Therefore, the acquisition time of spectra can be reduced to seconds allowing the real time tracking of the dynamics when turning flow on and off.[4] Here we use this particular property to observe the equilibration of the system, e.g. converging to a steady state. The enhancement of the signal in total internal reflection geometry can be described by Fresnel factors.[9] One would have to take them into

account when interfacial molecules are directly investigated by the spectral shape. As it is the magnitude of the signal, which correlates with the surface charge and is therefore of interest for our study, we integrate our spectra and do not focus on the spectral shape. Nevertheless, we note that Fig. 1b from the main text does not suggest significant changes of the spectral shape upon flow. For details on a relation between the spectral shape and Fresnel factors, we refer to other studies.[2,10-14]

Using total internal reflection geometry also requires consideration of the penetration depth of the evanescent fields that are generated at the interface. The interplay between the arising coherence length and the Debye length is complex.[5-7,15,16] If the penetration depth is shorter than the Debye length, the penetration depth would determine to which extent the $\chi^{(3)}$-term contributes to the signal. However, for our ionic strength in the millimolar range, the penetration depth of the evanescent field is much larger than the Debye length. Thus, we neglect the decay of the evanescent field and use the description of GONELLA et al.[6]

$$I_{\mathrm{SFG}} \sim E_{\mathrm{vis}}^2 E_{\mathrm{IR}}^2 \left|\chi^{(2)} + \chi^{(3)} \psi \cdot f_3\right|^2. \tag{S6}$$

Here $f_3$ describes a correction term for coherence, which for our ionic strength is $\sim 1$ and can, therefore, be neglected. The surface potential $\psi$ is described by the Graham equation:

$$\psi = 2 \frac{k_{\mathrm{B}} T}{e} \sinh^{-1}(2\pi\sigma\, \lambda_{\mathrm{B}} \lambda_{\mathrm{D}}) = 2 \frac{k_{\mathrm{B}} T}{e} \sinh^{-1}\left(\frac{e\sigma}{\sqrt{8 k_{\mathrm{B}} T N_{\mathrm{A}} \rho_{\mathrm{s}} \epsilon_0 \epsilon_{\mathrm{r}}}}\right), \tag{S7}$$

where $k_{\mathrm{B}}$ is the Boltzmann constant, $T$ the temperature, $e$ the elementary charge, $\sigma$ the number of charged surface groups, $\lambda_{\mathrm{B}}$ the Bjerrum length and $\lambda_{\mathrm{D}}$ the Debye length, $N_{\mathrm{A}}$ the Avogadro constant, $\rho_{\mathrm{s}}$ the background concentration in mol/m$^3$, $\epsilon_0$ the electric permittivity of vacuum, and $\epsilon_{\mathrm{r}}$ the relative permittivity of water.

As discussed in the main text, the flow-induced changes in ionic strength are much lower than the background ionic strength, which is why screening is excluded as a cause of the

observed changes. Therefore, and in accordance with other studies[4,17], we correlate the changes in the v-SFG response in this work solely to changes in the surface charge. This change in surface charge can influence not only the surface potential but also $\chi^{(2)}$ and thus both terms in equation (S5) in a complex manner [18]. For simplicity, we employ the following approximation

$$I_{SFG} \sim \psi^2. \tag{S8}$$

## Supplementary Note 2.

## Additional v-SFG Results

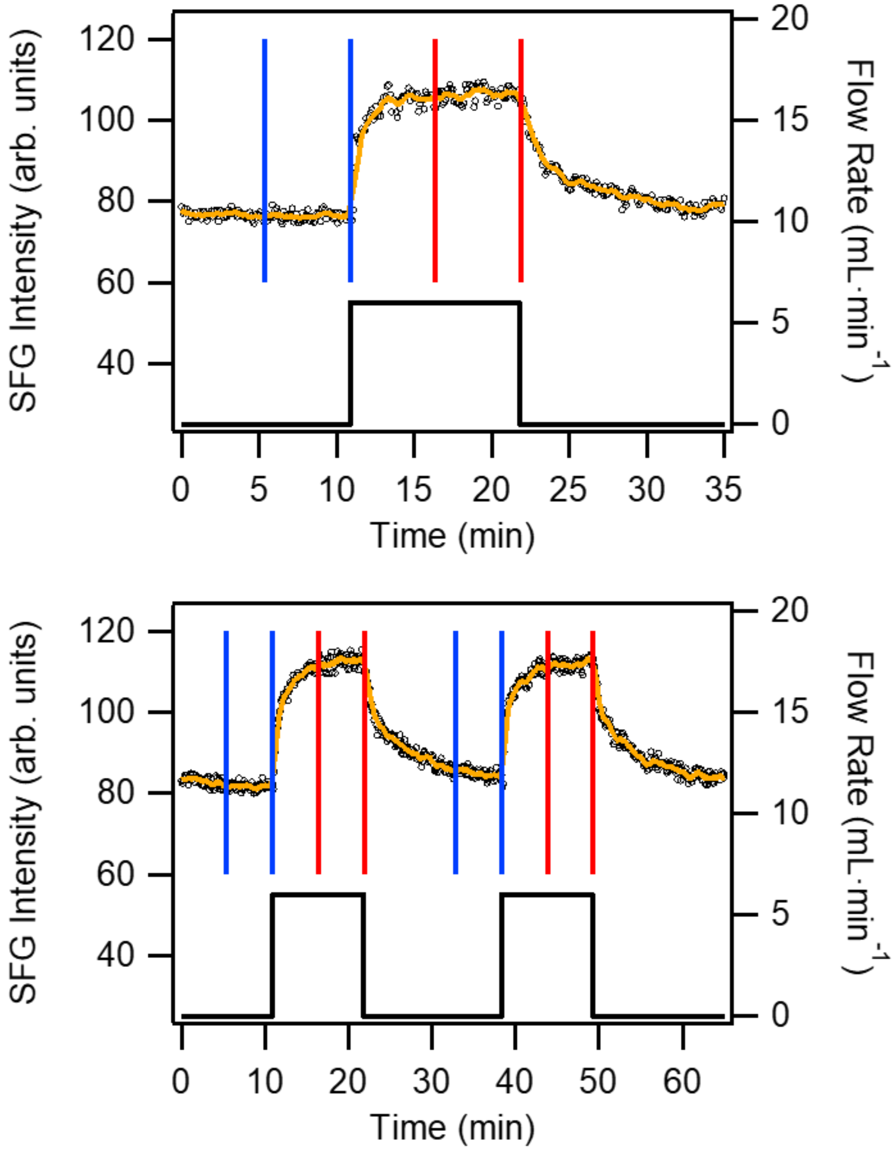


**Supplementary Figure 1. Exemplary time traces of the v-SFG signal with flow on/off cycles.** Similar to Fig. 1c in the main text, time traces of the integrated v-SFG signal (black circles) and a ten-point average (orange) as a guide for the eye are shown. The black curve shows the flow rate. The regimes marked by the blue and red lines are the flow-off and flow-on regimes, respectively. These regimes were used for calculating the relative increases in the v-SFG response due to flow. The change in the v-SFG intensity in these regimes is very low over time. Thus steady-state can be assumed.

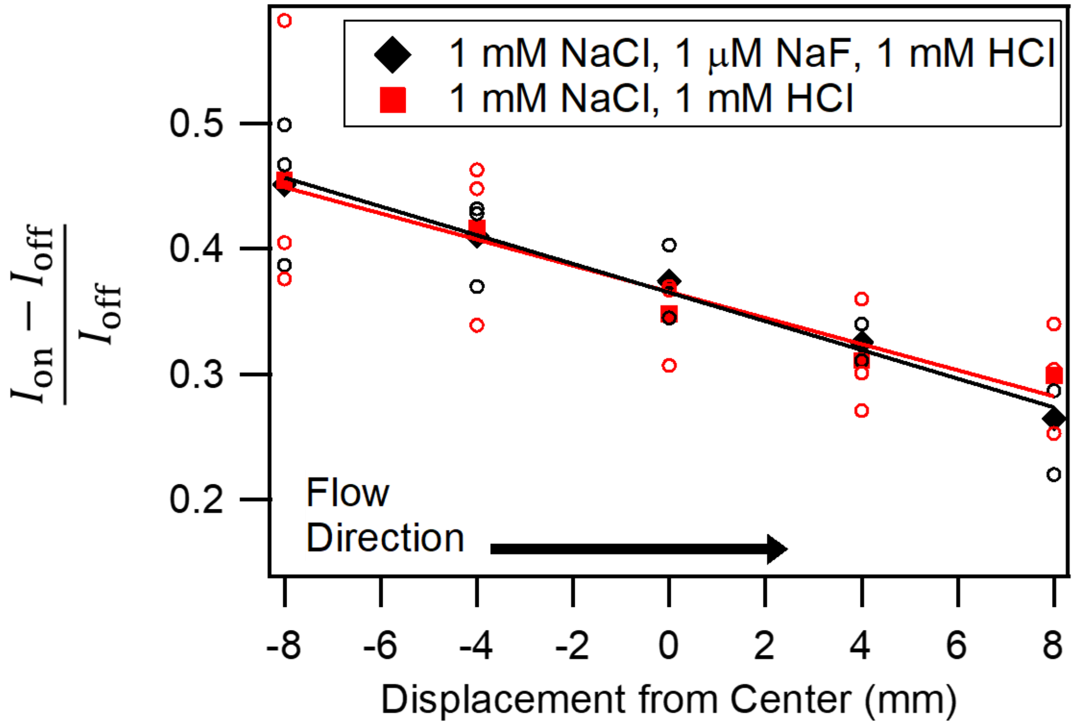


**Supplementary Figure 2. Gradient in the increase of the v-SFG intensity along the mineral surface.** At different positions, the relative increase in the v-SFG intensity at the $CaF_2$-water interface is calculated based on averaged steady-state spectra under flow-on and flow-off conditions as marked in Fig.1c of the main text and Supplementary Figure1. The open circles represent these ratios and the filled symbols are the mean of the ratios at the corresponding positions. The error of the individual ratios (open circles) due to small fluctuations within the steady-states of one flow-on/off cycle (see Fig1c of the main text and Supplementary Figure1) is negligible compared to the spread. The flow rate was 6mL/min. The solid lines are guides to the eye. The position is given as displacement from the center, with values increasing in the direction of flow, indicated by the arrow. The black data were already shown in Fig. 3 of the main text. By comparing the black and red data, it becomes clear that the addition of 1 μM NaF has no influence on the results as the points in black and red are within the spread of each other for every position.

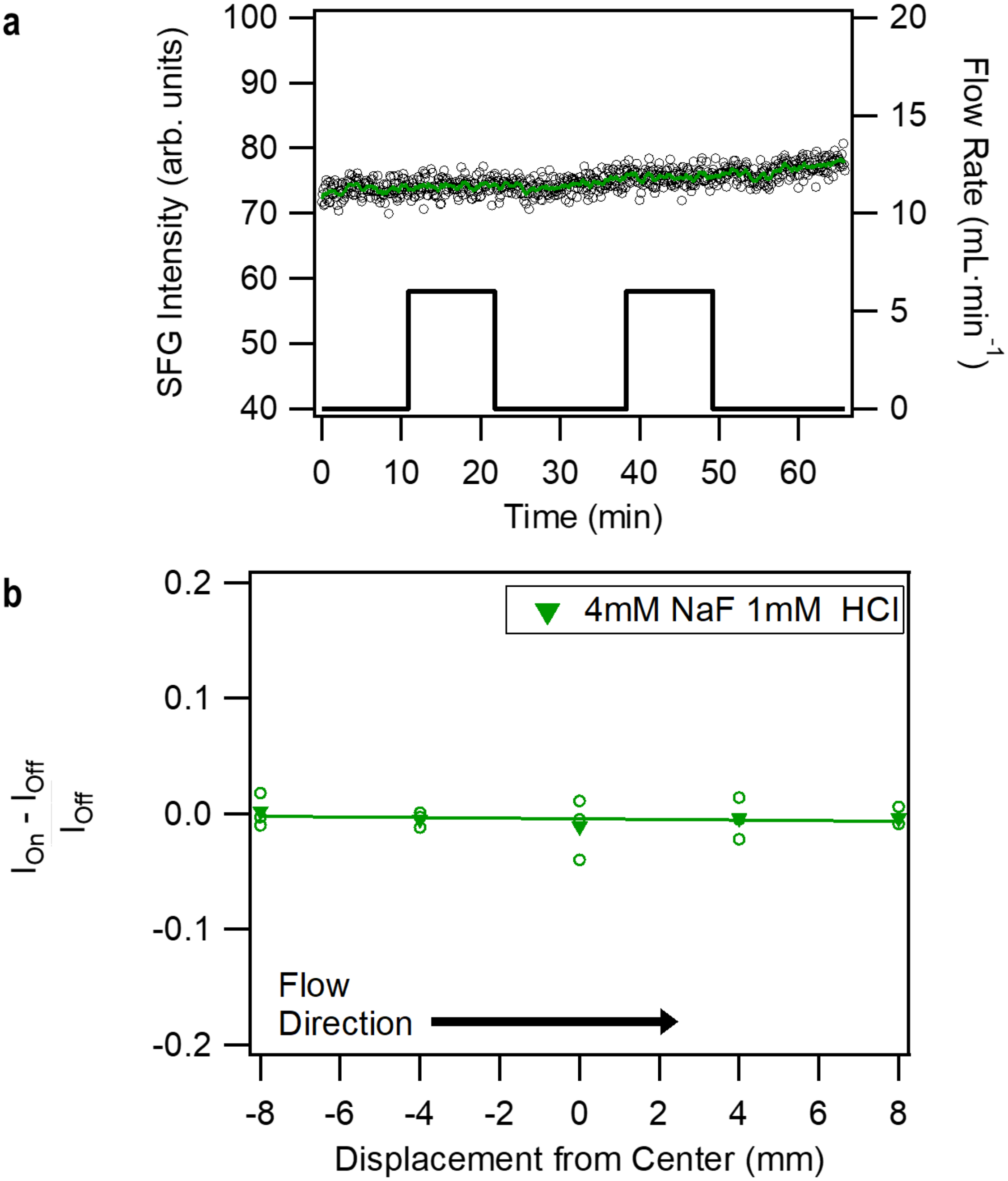


**Supplementary Figure 3. Experimental results at high fluoride concentrations.** (a) Exemplary time traces of the v-SFG signal with flow on/off cycles when using a 4 mM NaF solution in pH 3 HCl. (b) At different positions, the relative increase in the v-SFG intensity at the $CaF_2$-water interface is calculated based on averaged steady-state spectra under flow-on and flow-off conditions as marked in Fig1c of the main text and Supplementary Figure1. The open circles represent these ratios and the filled symbols are the mean of the ratios at the corresponding positions. The flow rate was 6mL/min. The solid line is a guide to the eye. The position is given as displacement from the center, with values increasing in the direction of flow, indicated by the arrow.

## Supplementary Note 3.

## Details of the Experimental Set up.

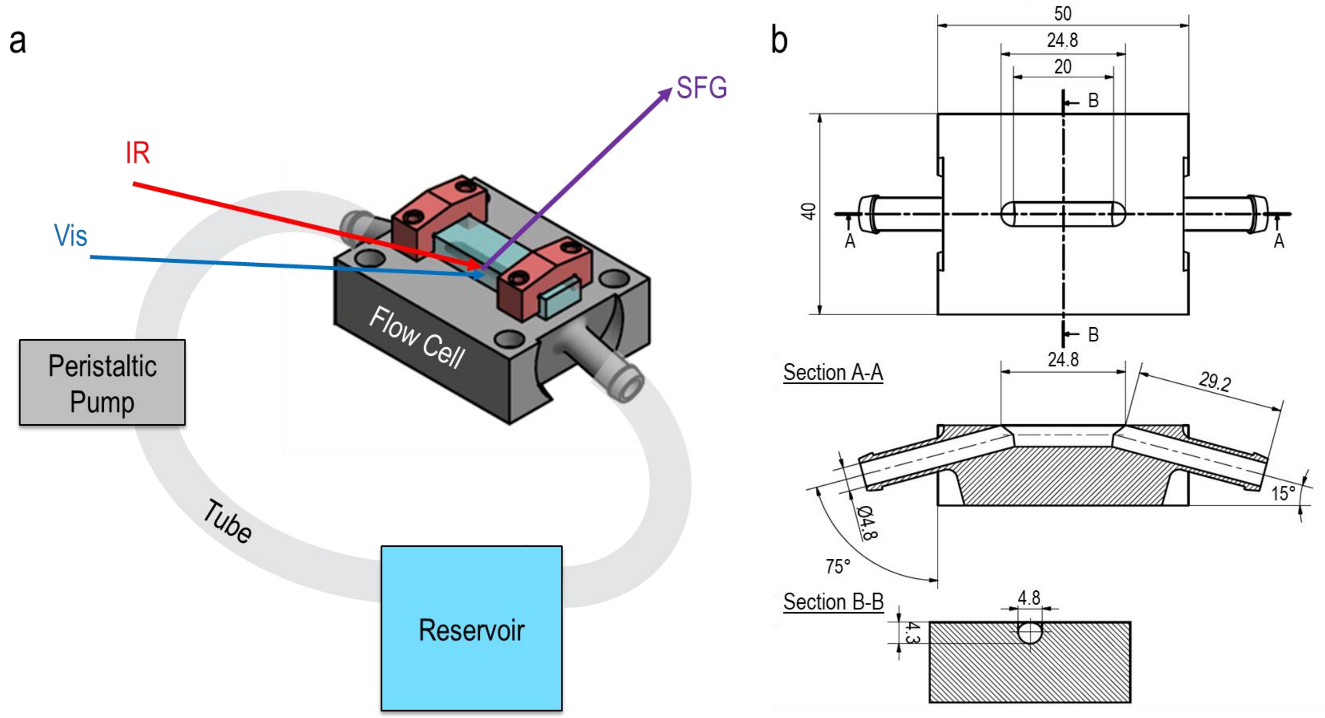


**Supplementary Figure 4. Illustration of Flow Set up.** (a) The flow cell has a flow channel with a rectangular $CaF_2$ prism mount on top. The Vis and IR laser pulse overlap at the center of the flow channel and generate SFG light in total internal reflection geometry. The flow cell is connected to a reservoir and a peristaltic pump. (b) Technical drawings of the flow cell with lengths in mm. Top panel shows a top view. A and B indicates the position of the cross sections in the middle and bottom panel, respectively.

## Supplementary Discussion 1.

### Full Poisson-Nernst-Planck-Stokes calculations

Solving the Poisson-Nernst-Planck equations with a finite element analysis requires a mesh size significantly smaller than the Debye length (~10 nm). We observed that the full experimental geometry has to be modeled, with a length scale of centimeters, to describe the experiments. The use of many more than $10^7$ mesh elements is computationally prohibitive. In this section, we will show that the results of small-scale Poisson-Nernst-Planck calculations are qualitatively similar and highlight a few minor quantitative deviations.

The set of equations that will be solved are the same as those in equations (3), (5)-(7) in the main text, but with the addition of the Poisson equation for the electric potential $\psi$, a conduction term added to the diffusion-advection equation, an extra electrostatic boundary condition relating the total surface charge density $e\sigma$ to the electric field over the surface normal $\mathbf{n}$. For completeness, a body force term $e\sum_i z_i\rho_i\ \boldsymbol{\nabla}\psi$, with $z_i$ the ion valency, has to be added to the Stokes equation. However, this term can be almost always neglected in pressure-driven flows. The set of equations is thus given by

$$\nabla^2\psi = -\frac{e}{\epsilon}\sum_i z_i\rho_i, \quad \text{(S9)}$$

$$\partial_t\mathbf{u} = -\boldsymbol{\nabla}p + \eta\nabla^2\mathbf{u} - e\sum_i z_i\rho_i\ \boldsymbol{\nabla}\psi, \quad \text{(S10)}$$

$$\partial_t\rho_i = D\nabla^2\rho_i - \boldsymbol{\nabla}\cdot(\rho_i\ \mathbf{u}) + D\frac{ez_i\rho_i}{k_BT}\ \boldsymbol{\nabla}\psi, \quad \text{(S11)}$$

$$\mathbf{n}\cdot\boldsymbol{\nabla}\psi = \frac{e\sigma}{\epsilon}, \quad \text{(S12)}$$

$$\psi(\pm\infty) = 0. \quad \text{(S13)}$$

In equilibrium this set of equations reverts back to regular Poisson-Boltzmann theory, that yields a diffuse layer of excess counter-ions, the electric double layer, near the charged surface. The decay length of the electric potential in this layer is the Debye length $\lambda_\mathrm{D} =$

$(8\pi\lambda_\mathrm{B}\rho_\mathrm{s})^{-1/2}$ where $\lambda_\mathrm{B}$ is the Bjerrum length and $\rho_\mathrm{s}$ the bulk concentration of an added 1:1 electrolyte. The geometry is scaled down to a cylindrical channel with length 5 μm and radius 0.15 $\mu$m. The length of the dissolving, charged surface in the middle of the channel is 2.5 $\mu$m. When including dissolution, a concentration profile of reactive ions forms, which breaks the translational invariance along the channel. When the concentration gradient over the Debye length is much larger than the concentration gradient over the channel, $\partial_r\rho \gg \partial_x\rho$, we can use the lubrication layer approximation[19]. This allows us to write the surface charge as $\sigma(x) = \Gamma\left(1 + k_\mathrm{ads}\rho(r=0,x)\, e^{\phi_0(\sigma(x))}/k_\mathrm{des}\right)^{-1}$, with $\phi_0=\psi_0\, e/k_\mathrm{B}T$ the dimensionless electric potential at the surface. We combine this result with the Gouy-Chapman result derived for the surface potential of flat plates finding $\phi(x) = 2\sinh^{-1}(2\pi\lambda_\mathrm{B}\lambda_\mathrm{D}\sigma(x))$. We compare the Gouy-Chapman surface potential with the numerically calculated surface potential in Supplementary Figure 5. Note that both the lubrication layer and Gouy-Chapman approximation work well at the $\mu m$ length scale, and the quality of the approximations is expected to increase at larger length scales.

When a pressure difference is applied over the channel, the electric boundary condition becomes of importance, as a streaming current is generated. In the experimental system, the fluid circulates, and inlet and outlet are connected, so boundary conditions representing a closed electric circuit are used: $\psi(x=\pm\infty)=0$. The resulting concentration profiles for Peclet numbers of one, two, and four times $3.5\cdot 10^6$ can be seen in Supplementary Figure 5. These calculations were repeated without any charge on the dissolving surface, but with the same surface reaction. The concentration profile in the middle stays the same with and without the conduction term.

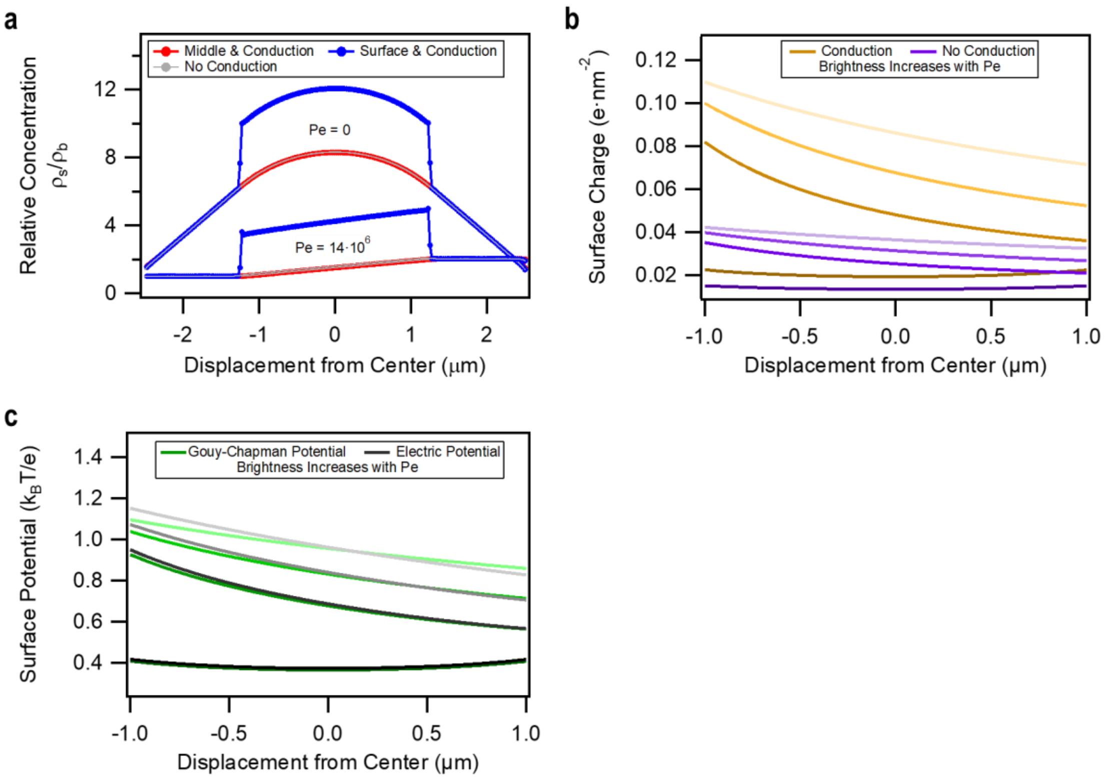


**Supplementary Figure 5. Comparison between Small Scale Calculations with and without a Conduction Term.** (a) The concentration on the central axis of the channel (red) and the concentration at the surface (blue) with a conduction term for the lowest and highest Peclet number investigated here, together with the corresponding profiles (gray) without conduction, which both overlap almost exactly with the red curves . The difference between the red and blue lines is due to electrostatic attraction between the surface and reactive ions, and can be accounted for by a Boltzmann factor $e^{\phi_0}$. (b) surface charge heterogeneity with conduction (orange) and without conduction (violet) for Peclet numbers 0, 1, 2 and 4 times $3.5 \cdot 10^6$ (dark to light). The large quantitative difference is again due to a change in concentration due to electrostatic attraction, but qualitatively the trends are the same. (c) Comparison between the electrostatic potential directly extracted from the numerical calculations with conduction (gray) and the surface potential as calculated by the Gouy-Chapman (green) from the same calculation for Peclet numbers 0, 1, 2 and 4 times $3.5 \cdot 10^6$ (dark to light).

The chief difference between these two concentration profiles is an increase of the concentration by a factor of $e^{\phi_0}$ at the surface. While this does not change any of the qualitative features of our model, namely a surface charge gradient induced by flow over a dissolving surface, it does affect the magnitude of this effect. This can be seen in Supplementary Figure 5b where we plot the obtained surface charge with and without conduction at the different flow rates. The difference between the two responses is significant (approximately a factor of five). This is mainly due to a rescaling of the equilibrium constant $K$ by a Boltzmann factor $K_{\mathrm{ch}} = K_{\mathrm{unch}} \mathrm{e}^{\phi_0}$, where $K_{\mathrm{ch}}$ is the equilibrium constant $k_{\mathrm{ads}}\rho_{\mathrm{b}}/k_{\mathrm{des}}$ with electrostatics and $K_{\mathrm{unch}}$ is the equilibrium constant without

electrostatics. As the equilibrium constant is an effective fit parameter used to reproduce the equilibrium surface potential $\psi_0$, this rescaling is effectively absorbed during our calculation in the main text. Note that the equilibrium surface potential is calculated using $K_{\mathrm{ch}}$ instead of $K_{\mathrm{unch}}$, which therefore accounts for the electric double layer. When the surface potential changes, the equilibrium constant also changes. This feature is neglected in the main text, however it only introduces minor quantitative deviations. Thus, the main conclusions from the numerical calculations with electrostatics included are that (i) the expression for equilibrated EDL's (Gouy-Chapman) is valid and hence non-equilibrium electrostatic phenomena can be neglected, and (ii) upon including electrostatics the same qualitative behavior is found as in the main text.

## Supplementary Discussion 2.

## Analytic one-dimensional model

As analytically solving the two-dimensional dissolution-diffusion-flow problem defined by equations (6)-(7) in the main text is intractable, we first convert the problem to an effective one-dimensional problem, which is analytically solvable. This one-dimensional problem can be solved while taking most experimental complications into account (back-reaction, a piece of the channel without a dissolving surface). However, the resulting analytic results are usually too complex to aid understanding of the system. Therefore, we introduce some further simplifications. The presented method can be straightforwardly extended to a parallel plate geometry.

We transform the two-dimensional diffusion-advection problem to a one-dimensional problem by radially integrating the diffusion-advection equation to obtain the average density $\bar{\rho}(x) = 2\pi \int \rho(x,r)\, r\, \mathrm{d}r/\pi R^2$. For convenience we drop the subscript F for the entirety of this section, but will still focus on the ion partaking both in charging and dissolution i.e. the fluoride concentration. In the regions without dissolution, we then find

$$\partial_t \bar{\rho} = D \partial_x^2\, \bar{\rho} - \partial_x \overline{u\rho}. \tag{S14}$$

Whereas the diffusive term is straightforwardly integrated, the calculation of the advective term is more complicated. This is because it results in a density-weighted velocity $\overline{u\rho}$=$2\pi \int u_x(r) \rho(r,x)\, r\, \mathrm{d}r/\pi R^2$, which requires the knowledge of $\rho(x,r)$ as a function of the radial coordinate to calculate. In the narrow-channel limit $\frac{\delta}{R} \gg 1$, however, the approximation $\rho(x,r) = \bar{\rho}(x)$ is valid as shown in the main text, and the radially averaged advection-diffusion equation simply becomes:

$$\partial_t \bar{\rho} = D \partial_x^2\, \bar{\rho} - \bar{u} \partial_x \bar{\rho}. \tag{S15}$$

In the opposite limit of a thin boundary layer ($\frac{\delta}{R} \ll 1$) the integral $\overline{u\rho}$ does not factorize as there is a significant radial variation in the concentration $\rho(x,r)$. A qualitative approximation can be made in the limit of high Peclet ($\mathrm{Pe} \gg 1$) by replacing the average velocity $\bar{u}$ by the effective boundary transfer velocity $h \propto \bar{u}^{2/3}$, as will be derived in Supplementary Discussion 2. The boundary transfer velocity accounts for the effect that at high flow velocities the ions released from the surface do not have time to spread out over the length of the entire radius by diffusion. This lowers the effective flow velocity ($h \propto \bar{u}^{2/3}$) as the velocity near the surface of the channel is lower than at the center.

Continuing with the narrow channel limit $\frac{\delta}{R} \gg 1$ , where $\overline{u\rho} = \bar{u}\bar{\rho}$, the integration of equation (S15) in the region with a dissolving surface (represented by the source term **J**) is straightforward and results in

$$\partial_t \bar{\rho} \approx D\partial_x^2 \bar{\rho} - \bar{u}\partial_x \bar{\rho} + 2n\frac{k_{\mathrm{dis}} - k_{\mathrm{prec}}\rho_{\mathrm{b}}}{R} - 4n\frac{\mathrm{k_{prec}}}{R}(\bar{\rho} - \rho_{\mathrm{b}}), \qquad \text{(S16)}$$

where the dissolution term was substituted for the radial concentration gradient at the boundary $\mathbf{n} \cdot \mathbf{J} = D\partial_r \rho$. We linearized the precipitation in the density $k_{\mathrm{prec}}\rho^n \approx k_{\mathrm{prec}}\rho_{\mathrm{b}} - n\, k_{\mathrm{prec}}(\rho - \rho_{\mathrm{b}})$ to ensure that the equation remains a linear, solvable differential equation. Here $n$ is the stochiometric constant relating the number of ions released per uncharged unit. Note that these equations are approximations valid in the limit $\frac{\delta}{R} \ll 1$.

From differential equations equation (S15) and equation (S16) we can construct analytic solutions for simple geometries involving patches of non-dissolving and dissolving channels. For instance, in a channel with a dissolving surface in the middle separated by two regions without dissolution to two bulk reservoirs, we would have three differential equations, namely two patches without dissolution and one patch with dissolution. For the dissolving surface, we would use equation (S16), and for the regions without a dissolving surface, equation (S15) is used. At the edge of each discontinuity, the boundary condition $\bar{\rho}_n(x_n) = \rho_n$ is used, where $x_n$ is the boundary between geometry elements $n$ and $n+1$, $\bar{\rho}_n$ denotes

the solution to the $n$th differential equation. The boundary concentration $\rho_n$ is an unknown concentration that can be fixed by using that the average concentration $\bar{\rho}_n$ at each boundary is continuous $\bar{\rho}_n(x_n) = \bar{\rho}_{n+1}(x_n)$, which is true as long as the radius $R(x)$ is continuous. This then gives $n-1$ equalities for $n+1$ unknown concentrations. The two unknown concentrations are located at the edges of the model system (for instance, the edge of the bulk reservoirs). In this work, the boundary condition $\bar{\rho}_1(x_0) = \bar{\rho}_N(x_N) = \rho_b$, with $x_0$ being the leftmost system boundary and $x_N$ being the rightmost system boundary, was always used. While this set of equations is always analytically solvable (and is straightforwardly extended to, for instance, multiple reactive patches) the resulting expressions are often so complicated to the point of being illegible, even with only three spatial regions (two non-dissolving patches and a channel with only a dissolving surface). The solutions are computationally accessible, so the solutions can be straightforwardly plotted to gain insight without having to resort to numerical calculations. Here we will consider the solution in a simplified case, leading to the result shown in the main text. We consider a geometry with only one part; a dissolving channel of length $2L$, with radius $R$, and neglect the precipitation term. We chose not to add the two non-dissolving walls because of the additional complexity in the final expressions. The resulting expression is

$$\frac{\rho(x)}{\rho_{\mathrm{b}}} = 1 + 2\frac{\Delta\rho_{\max}}{\mathrm{Pe}}\left(\frac{2x}{L} + \left(1 + e^{\,\mathrm{Pe}} - 2e^{\frac{\mathrm{Pe}}{2}\left(1+\frac{x}{L}\right)}\right)\left(\mathrm{Coth}\left(\frac{\mathrm{Pe}}{2}\right) - 1\right)\right) \qquad \text{(S17)}$$

which, in the limit of low and high Peclet number, simplifies to equation (8) in the main text. Here $\Delta\rho = k_{\mathrm{dis}}L^2/DR\rho_{\mathrm{b}}$ and $\mathrm{Pe} = 2\bar{u}L/D$. It can be seen in Figure 7 in the main text that this analytic result matches numerical results in the narrow channel limit ($\delta/R \gg 1$)

In principle, this one-dimensional model is fully solvable for any radially symmetric geometry with continuous radius $R(x)$ and constant radial concentration $\rho(x,r) = \bar{\rho}(x)$. In practice, the resulting expressions, while valid, become so complex that they are useless for insight and even become computationally heavy to plot and manipulate. Highly simplified analytic models lose quantitative predictive power but incorporate all the fundamental

physics in the problem: dissolution, advection, and reactions, and qualitatively describes how they balance each other.

## Supplementary Discussion 3.

## Diffuse boundary layer

In this section we summarize the derivation of the diffuse boundary layer as presented elsewhere[20]. A diffuse boundary layer is the concentration profile that forms near a dissolving surface when lateral advection is so strong that there is not enough time for radial diffusion to occur. As in the previous section, we drop the subscript $F$ on the density $\rho$, but we are still explicitly solving for the fluoride concentration.

Observing that at high Peclet numbers lateral diffusion can be neglected, the diffusion-advection equation can be written in stationary state as

$$\partial_t \rho = D\nabla^2 \rho - \nabla \cdot (\mathrm{u}\rho) \approx \frac{D}{r}\partial_r(r\partial_r \rho) - u_x(r)\partial_x \rho = 0. \tag{S18}$$

Now we consider a very long channel with a fully developed laminar flow profile with a laterally *constant* surface concentration $\rho_\mathrm{m}$, for which the boundary conditions can be written as

$$\begin{cases} \rho(\pm\infty, r) = \rho_{\mathrm{b},} \\ \rho(x, R) = \rho_\mathrm{m}, \\ \partial_r \rho(x, 0) = 0. \end{cases} \tag{S19}$$

This is traditionally known as the Graetz problem[20-22]. The assumption of a constant surface concentration is not valid in the model considered in the main text. However, the present analysis is concerned with describing the concentration profile some distance $\delta$ from the

surface. We will solve differential equation (S18) together with boundary conditions equation (S19) using the Ansatz that the solution is self-similar, and that the concentration profile $\rho(x, r)$ that depends on two variables can be described by a function of a single self-similar variable $\eta$

$$\rho(x, r) = f(\eta). \tag{S20}$$

The existence of a self-similar solution is suggested[23] because it is not possible to define a dimensionless lateral position $X$ using only the lateral position and velocity, $x$ and $u_x$, and hence the concentration should be reducible to a one parameter equation of $\eta \propto (R - r)/\delta(x)$. Now we define the dimensionless, self-similar, parameters

$$\eta = \frac{R - r}{\delta(x)} = (R - r)\left(\frac{4\,\bar{u}}{9DR(x + H)}\right)^{\frac{1}{3}}, \tag{S21}$$

$$\zeta = \frac{\delta(x)^3}{R^3} = \frac{9D(x + H)}{4\bar{u}R^2}, \tag{S22}$$

$$\Theta = \frac{\rho - \rho_{\mathrm{m}}}{\rho_{\mathrm{b}} - \rho_{\mathrm{m}}}. \tag{S23}$$

and using this we rewrite equation (S19) to[20]

$$\frac{\partial^2\Theta}{\partial\eta^2} + 3\eta^2\frac{\partial\Theta}{\partial\eta} - 3\eta\zeta^{\frac{1}{3}}\frac{\partial\Theta}{\partial\zeta^{\frac{1}{3}}} = \left(\frac{3}{2}\eta^3\zeta^{\frac{1}{3}} + \frac{\zeta^{\frac{1}{3}}}{1 - \eta^3\zeta^{\frac{1}{3}}}\right)\frac{\partial\Theta}{\partial\eta} - \frac{3}{2}\eta^2\zeta^{\frac{2}{3}}\frac{\partial\Theta}{\partial\zeta^{\frac{1}{3}}}. \tag{S24}$$

We find the dimensionless boundary conditions

$$\begin{cases} \Theta(\eta = \infty) = 1, \\ \Theta(\eta = 0) = 0. \end{cases} \tag{S25}$$

The solution to equation (S24) can be expanded as function of $\zeta = \delta/R$

$$\Theta = \Theta_0(\eta) + \zeta^{\frac{1}{3}}\Theta_1(\eta) + \zeta^{\frac{2}{3}}\Theta_2(\eta) + O(\zeta), \tag{S26}$$

where at high Peclet number we only consider the dominant zero-order term, also known as the Lévêque approximation

$$\frac{\mathrm{d}^2\Theta_0}{\mathrm{d}\eta^2} + 3\eta^2 \frac{\mathrm{d}\Theta_0}{\mathrm{d}\eta} = 0. \tag{S27}$$

This is approximation is equal to only considering the first order term of the velocity expansion $u(r) \approx 4\bar{u}(1 - r/R) + O((1 - r/R)^2)$ [21] close to $r = R$, and hence is valid in the limit of high Peclet number where $\delta/R \ll 1$. Solving for $\Theta_0$ we find the Lévêque solution

$$\Theta_0(\eta) = C^{-1} \int_0^{\eta} e^{-s^3} \, \mathrm{d}s, \tag{S28}$$

$$C = \int_0^{\infty} e^{-s^3} \, \mathrm{d}s = \Gamma\left(\frac{4}{3}\right) \approx 0.9. \tag{S29}$$

with $\Gamma$ the Euler gamma function. Plotting the concentration profile in Supplementary Figure 6 as a function of the radial coordinate at $x = 0$ we see that the radial concentration profile is very well approximated by the Lévêque solution. In Supplementary Figure 7 we plot the

radial position $R - r$ at which the concentration satisfies $1.9 < \frac{\rho_{\mathrm{F}}(r)}{\rho_{\mathrm{b,F}}} < 2.1$ together with $\delta(x)$ as function of $(x + H)^{1/3}$. We find that the boundary layer thickness in our numerical calculations is linear in$(x + H)^{1/3}$ as expected.

Thus, for $x = 0$ and at a flow rate of 1 mL/min we find that the typical boundary layer thickness at which the radial concentration has decayed by 90% of its surface value is approximately $\delta \approx 0.4$ mm.

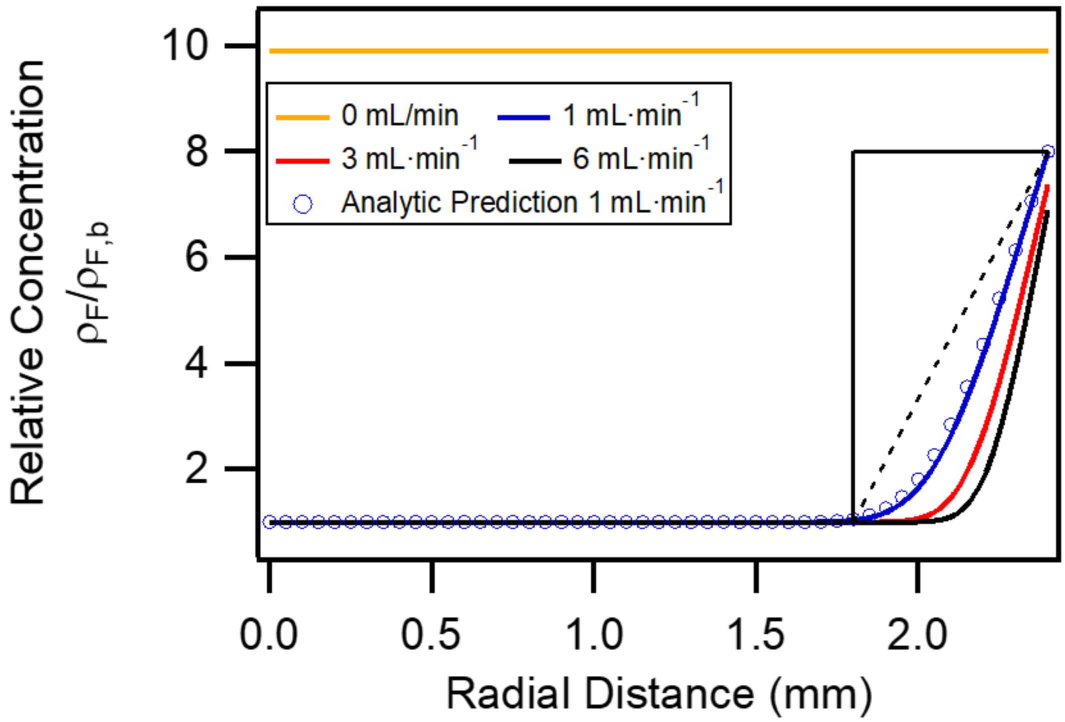


**Supplementary Figure 6. Radial Concentration Profiles.** The radial concentration profile of reactive ions in the center of the channel at different flow speeds are shown. The data were obtained from full numerical calculations as described in the main text. It can be seen that close to the surface, the concentration profile is well described by a linear concentration profile with a flow-dependent length scale. Circles are the analytically predicted diffuse boundary layer at the lowest flow rate according to equation (S28) with the maximum density $\rho_m$ fit parameter.

While the behavior of the concentration profile at some distance from the channel wall is thus correctly described by diffuse boundary layer theory, the near-surface ($R - r \ll \delta$) profile deviates significantly from the Lévêque solution as the assumption of a constant surface concentration is incompatible with the macroscopic concentration gradient, which is the main object of interest in the main text. Thus, while superficially similar to a cuberoot

scaling, the surface concentration $\rho(x,R)$ does not follow the power law $(x+H)^{1/3}$. To estimate the influence of the diffuse boundary layer on the surface concentration $\rho(x,R)$ we insert an approximation for the radial concentration profile (S34, S36) into our analytic calculation as presented in Supplementary Discussion 2, which allows us to calculate $\rho_{\rm m} = \rho(x,R)$. Note that this combination of diffuse boundary layer theory and the radially integrated diffusion-advection equation does not result in a fully self-consistent theory. However it does allow us to estimate the change in surface concentration with regard to flow-rate.

The fact that the diffuse boundary layer is localized near the channel wall where the velocity is low implies that the effective velocity for the transport of ions is much lower than the channel-averaged velocity. To estimate the effect of the small boundary layer thickness we need an expression for $\rho(x,r)$ such that the integral $\int_0^R \rho(r)u(r)r\,\mathrm{d}r$ can be evaluated, as required for S14. Inspecting the numerically obtained concentration profiles in Supplementary Figure 5 we see that the it can be approximated by the simple form

$$\rho(r,x) = \begin{cases} \rho_{\rm b} & \text{if} \quad r < R-\delta(x), \\ \rho_{\rm b} + \dfrac{\rho_{\rm m}-\rho_{\rm b}}{\delta(x)}(r-R+\delta(x)) & \text{if} \quad R-\delta(x) < r < R. \end{cases} \qquad \text{(S30)}$$

With this approximation the divergence of the density weighted velocity $\overline{u\rho}$, as defined in equation (S14), can be straightforwardly found

$$\partial_x\,\overline{u\rho} = \partial_x\left(\bar{u}\,\frac{4\delta^2}{3R^2}(\rho_{\rm m}-\rho_{\rm b})\right) + O\left(\frac{\delta^3}{R^3}\right) \approx \partial_x\,\frac{4\bar{u}\delta}{3R}\,\bar{\rho}. \qquad \text{(S31)}$$

From this we can define the effective density weighted velocity $h$, also known as the boundary transfer velocity[22]. The boundary transfer velocity is given by $h = 4\bar{u}\delta/3R$ when the surface concentration is much larger than the bulk concentration ($\rho_m \gg \rho_{\rm b}$), in which

case $\bar{\rho} = \rho_{\mathrm{m}}\delta/R$, and the gradient in the surface concentration is larger than the gradient in the boundary thickness ($\rho_{\mathrm{m}}^{-1}\partial_x\rho_{\mathrm{m}} \gg \delta^{-1}\partial_x\delta$). As seen from S28 the boundary thickness satisfies $\delta \propto \bar{u}^{-1/3}$ and thus the effective transfer velocity scales as

$$h = \bar{u}\frac{4\delta}{3R} \propto \bar{u}^{2/3}. \quad \text{(S32)}$$

From this it can be straightforwardly seen that the Sherwood number Sh, which is the dimensionless number comparing the boundary transfer rate with the diffusion rate, scales as Sh$= 2Lh/D \propto \mathrm{Pe}^{2/3}$ [20,22]. While the effective advection rate scaling by a power of 1/3 with the channel-averaged advection rate is a well-known result for a channel wall, kept at a *constant* concentration[20-22], here we show that a phenomenological model for a dissolving channel with a *heterogeneous* surface concentration gives rise to a power of 2/3.

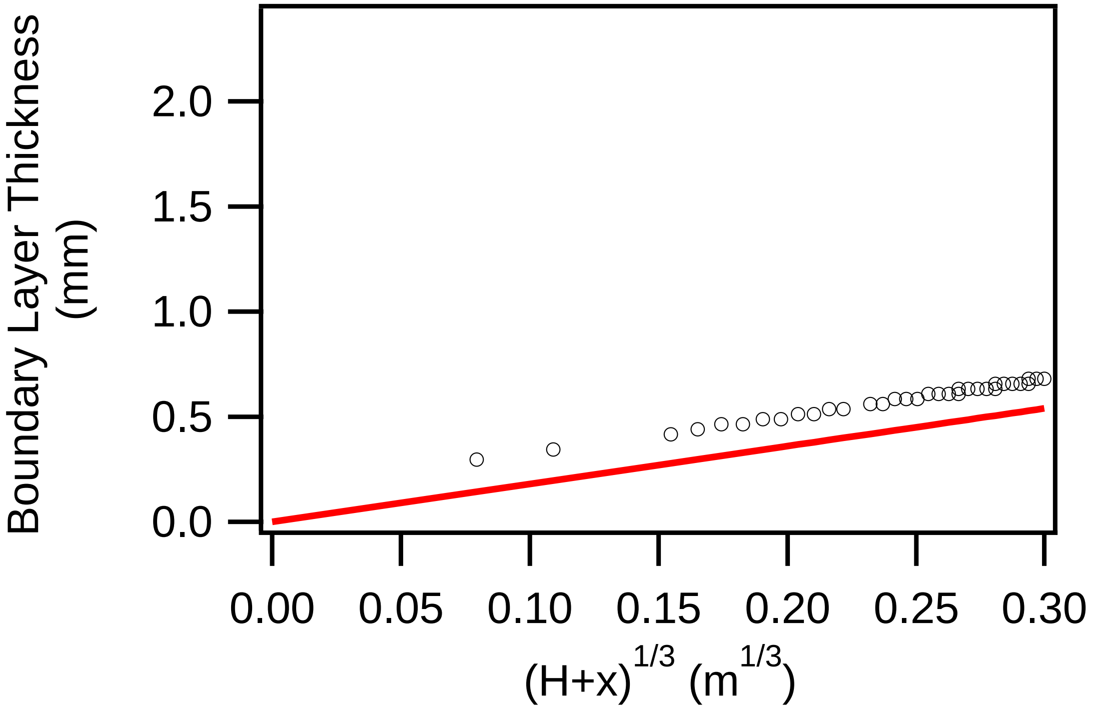


**Supplementary Figure 7. Verification cube root scaling diffuse boundary layer thickness.** The radial position at which the concentration is between $1.9 < \rho/\rho_b < 2.1$ (data points) against the cube root of the lateral position $(H + x)^{1/3}$, plotted from the $x = -H$ to $x = H$. It can be clearly seen that the numerical data is linear in the cube root of the position $(H + x)^{1/3}$. The $y$-axis scale is between $0 < r < R$.

The Sherwood number replacing the Peclet number and scaling as a power smaller than 1 of the latter captures an important aspect of our experiment, namely that the effective advection rate over the boundary layer is much lower than the channel-averaged advection rate. This causes the sharp transition between the flow and no-flow concentrations seen in Supplementary Figure 7b to broaden significantly to larger Peclet number. As the experimental Peclet number is $\mathrm{Pe} \approx 10^5$, the Sherwood number is lower by almost two orders of magnitude $\mathrm{Sh} \approx 10^3$. Combined with the precipitation reaction, which increases the dissolution rate at lower surface concentration, the lower effective advective transfer rate $h$ explains why there is still a significant change of concentration with flow while the limit of large Peclet number has been reached in our experiments. We stress that the scaling relation between surface concentration and flow rate in the case of a fully developed diffuse boundary layer is an order-of-magnitude estimation and that we have not found a regime in which it is quantitatively accurate.

## Supplementary References


1 Lambert, A. G., Davies, P. B. & Neivandt, D. J. Implementing the Theory of Sum Frequency Generation Vibrational Spectroscopy: A Tutorial Review. *Appl. Spectrosc. Rev.* **40**, 103-145 (2005).

2 Morita, A. *Theory of Sum Frequency Generation Spectroscopy*. Vol. 35 (Springer, 2018).

3 Bonn, M., Nagata, Y. & Backus, E. H. G. Molecular structure and dynamics of water at the water-air interface studied with surface-specific vibrational spectroscopy. *Angew. Chem. Int. Ed. Engl.* **54**, 5560-5576 (2015).

4 Lis, D., Backus, E. H. G., Hunger, J., Parekh, S. H. & Bonn, M. Liquid flow along a solid surface reversibly alters interfacial chemistry. *Science* **344**, 1138-1142 (2014).

5 Jena, K. C., Covert, P. A. & Hore, D. K. The Effect of Salt on the Water Structure at a Charged Solid Surface: Differentiating Second- and Third-order Nonlinear Contributions. *J. Phys. Chem. Lett.* **2**, 1056-1061, doi:10.1021/jz200251h (2011).

6 Gonella, G., Lütgebaucks, C., de Beer, A. G. F. & Roke, S. Second Harmonic and Sum-Frequency Generation from Aqueous Interfaces Is Modulated by Interference. *J. Phys. Chem. C* **120**, 9165-9173, doi:10.1021/acs.jpcc.5b12453 (2016).

7 Schaefer, J., Gonella, G., Bonn, M. & Backus, E. H. G. Surface-specific vibrational spectroscopy of the water/silica interface: screening and interference. *Phys. Chem. Chem. Phys.* **19**, 16875-16880 (2017).

8 de Beer, A. G. F., Campen, R. K. & Roke, S. Separating surface structure and surface charge with second-harmonic and sum-frequency scattering. *Phys. Rev. B* **82**, doi:10.1103/PhysRevB.82.235431 (2010).

9 Löbau, J. & Wolfrum, K. Sum-frequency spectroscopy in total internal reflection geometry: signal enhancement and access to molecular properties. *J. Opt. Soc. Am. B* **14**, 2505-2512, doi:10.1364/JOSAB.14.002505 (1997).

10 Backus, E. H. G., Garcia-Araez, N., Bonn, M. & Bakker, H. J. On the Role of Fresnel Factors in Sum-Frequency Generation Spectroscopy of Metal–Water and Metal-Oxide–Water Interfaces. *J. Phys. Chem. C* **116**, 23351-23361, doi:10.1021/jp306273d (2012).

11 Sun, S., Schaefer, J., Backus, E. H. G. & Bonn, M. How surface-specific is 2nd-order non-linear spectroscopy? *J. Chem. Phys* **151**, 230901, doi:10.1063/1.5129108 (2019).

12 Shen, Y. Optical second harmonic generation at interfaces. *Annu. Rev. Phys. Chem.* **40**, 327-350 (1989).

13 Wei, X., Hong, S.-C., Zhuang, X., Goto, T. & Shen, Y. R. Nonlinear optical studies of liquid crystal alignment on a rubbed polyvinyl alcohol surface. *Phys. Rev. E* **62**, 5160-5172, doi:10.1103/PhysRevE.62.5160 (2000).

14 Zhuang, X., Miranda, P. B., Kim, D. & Shen, Y. R. Mapping molecular orientation and conformation at interfaces by surface nonlinear optics. *Phys. Rev. B* **59**, 12632-12640, doi:10.1103/PhysRevB.59.12632 (1999).

15 Jena, K. C. & Hore, D. K. Variation of Ionic Strength Reveals the Interfacial Water Structure at a Charged Mineral Surface. *J. Phys. Chem. C* **113**, 15364-15372, doi:10.1021/jp905475m (2009).

16 Hore, D. K. & Tyrode, E. C. Probing Charged Aqueous Interfaces Near Critical Angles: Effect of Varying Coherence Length. *J. Phys. Chem. C*, doi:10.1021/acs.jpcc.9b05256 (2019).

17 Xi, Y. *et al.* Flow Alters the Interfacial Reactions of Upconversion Nanocrystals Probed by In Situ Sum Frequency Generation. *Adv. Mater. Interfaces* **7**, 1902046 - 1190205 (2020).

18 Wen, Y. C. *et al.* Unveiling Microscopic Structures of Charged Water Interfaces by Surface-Specific Vibrational Spectroscopy. *Phys. Rev. Lett.* **116**, 016101, doi:10.1103/PhysRevLett.116.016101 (2016).

19 Malgaretti, P., Janssen, M., Pagonabarraga, I. & Rubi, J. M. Driving an electrolyte through a corrugated nanopore. *J. Chem. Phys* **151**, 084902 (2019).

20 Newman, J. Extension of the Leveque Solution. *J. Heat Transfer.* **91**, 177-178 (1969).

21 Belhocine, A. & Wan Omar, W. Z. Similarity solution and Runge Kutta method to a thermal boundary layer model at the entrance region of a circular tube: The Lévêque Approximation. *Rev. Cient.* **1**, 1-26, doi:10.14483/23448350.12506 (2018).

22 Bird, R. B., Stewart, W. E. & Lightfoot, E. N. *Transport Phenomena*. (Wiley, 2006).

23 Barenblatt, G. I., Barenblatt, G. I. & Isaakovich, B. G. *Scaling, self-similarity, and intermediate asymptotics: dimensional analysis and intermediate asymptotics*. (Cambridge University Press, 1996).
